# Bioinformatics analysis of the pathogenic link between Epstein-Barr virus infection, systemic lupus erythematosus and diffuse large B cell lymphoma

**DOI:** 10.1038/s41598-023-33585-2

**Published:** 2023-04-18

**Authors:** Qian-Ying Zhu

**Affiliations:** Department of Laboratory Medicine, The Eighth Affiliated Hospital, Sun Yat-sen University, Shenzhen, 518003 People’s Republic of China

**Keywords:** Computational biology and bioinformatics, Immunology, Oncology, Pathogenesis, Rheumatology

## Abstract

Epstein-Barr virus (EBV) is a risk factor for diffuse large B-cell lymphoma (DLBCL) and systemic lupus erythematosus (SLE). While prior research has suggested a potential correlation between SLE and DLBCL, the molecular mechanisms remain unclear. The present study aimed to explore the contribution of EBV infection to the pathogenesis of DLBCL in the individuals with SLE using bioinformatics approaches. The Gene Expression Omnibus database was used to compile the gene expression profiles of EBV-infected B cells (GSE49628), SLE (GSE61635), and DLBCL (GSE32018). Altogether, 72 shared common differentially expressed genes (DEGs) were extracted and enrichment analysis of the shared genes showed that p53 signaling pathway was a common feature of the pathophysiology. Six hub genes were selected using protein–protein interaction (PPI) network analysis, including CDK1, KIF23, NEK2, TOP2A, NEIL3 and DEPDC1, which showed preferable diagnostic values for SLE and DLBCL and involved in immune cell infiltration and immune responses regulation. Finally, TF-gene and miRNA-gene regulatory networks and 10 potential drugs molecule were predicted. Our study revealed the potential molecular mechanisms by which EBV infection contribute to the susceptibility of DLBCL in SLE patients for the first time and identified future biomarkers and therapeutic targets for SLE and DLBCL.

## Introduction

Epstein-Barr virus (EBV) is considered to be one of eight human herpesviruses that contain protein capsids around the double-stranded linear DNA genome^1^. EBV infects over 90% of people worldwide. The oral cavity is assumed to be the site of primary EBV infection^2^. Lymphocytes and epithelial cells make up the majority of host cells for EBV^3^. In adolescents, primary EBV infection often causes infectious mononucleosis (IM). Several autoimmune disorders, including systemic lupus erythematosus (SLE), have been linked to EBV^4^. In addition, as the first tumorigenic virus to be identified, EBV causes approximately 200,000 new instances of cancer each year, comprising cancers originating from B cells like Burkitt lymphoma and diffuse large B-cell lymphoma (DLBCL)^5^.

SLE is an autoimmune disorder that usually occurs among women of childbearing age with multi-systemic involvement^6^. The phenotypes of SLE can vary from minor mucocutaneous signs to serious central nervous or kidney damage, which results in a high risk of death for patients^7^. The characteristic feature of SLE is the occurrence of autoantibodies against nuclear antigens (ANA), which can be detected up to 10 years before disease onset^8^. SLE is considered to be brought on by a confluence of hereditary and environmental factors. Among environmental factors, viral infection, especially EBV infection, is closely related to SLE^9^. Previous studies have shown that, compared to healthy controls, higher viral loads and elevated titers of antibodies against EBV are detected among SLE patients^10^. Moreover, a growing body of research suggests that EBV-infected B cells may become resistant to apoptosis, leading to the proliferation, activation and antibody production of autoreactive B cells, which may result in tissue damage for SLE^11^. However, the underlying genetic molecular mechanism of EBV infection on the development of SLE is still not fully elucidated.

DLBCL refers to diffuse growth tumors with nuclei greater than 2 normal lymphocytes. It is the most prevalent kind of non-Hodgkin's lymphoma, making up roughly 40% of all B-cell lymphomas^12^. The R-CHOP therapy can cure approximately 60% of DLBCL. However, many patients still develop resistant to this treatment or experience a recurrence and eventually die^13^. At present, the etiology of DLBCL is not particularly clear, which is usually associated with gene abnormalities, EBV infection and other reasons^14^. In 2016, the World Health Organization classified EBV-associated DLBCL as a new subtype of DLBCL (EBV + DLBCL, NOS)^15^. Although a number of researches have demonstrated that EBV play extremely crucial roles in the process of inducing malignant transformation of lymphocytes^16,17^, the specific molecular mechanism of EBV regulating B cell signaling pathway still needs to be further studied.

Some studies have found an elevated incidence of malignancies, notably lymphoma, in people with SLE^18^. DLBCL composes 37–62% of all lymphomas identified in SLE^19^, but there is currently a lack of knowledge on the pathophysiology between DLBCL and SLE. Several determinants could be behind this altered risk, such as genetic factors, immunologic derangements and viruses, among which EBV infection is suggested to be the link between SLE and DLBCL^20^. Recent studies have shown that persistent EBV infection in SLE patients can promote malignant transformation of B cells due to the use of immunosuppressive drugs and other reasons^21^. However, molecular mechanisms underlying the contribution of EBV infection to the development of DLBCL in SLE patients are yet unknown.

In this study, we attempted to identify the shared gene signatures between EBV infection, SLE and DLBCL and explore the possible biological effect of EBV infection to the pathogenesis of DLBCL in the context of SLE. Firstly, we employed three datasets in this investigation to explore the biological link between EBV infection, SLE, and DLBCL. GSE49628, GSE61635, and GSE32018 were chosen from the Gene Expression Omnibus (GEO) database for EBV infection, SLE, and DLBCL, respectively. We first identified the differentially expressed genes (DEGs) in each dataset before identifying the common DEGs in these three datasets. To comprehend genome-based biological processes, shared DEGs were employed as the key experimental genes throughout the study, including gene ontology (GO) analyses, pathway enrichment analyses, and protein–protein interaction (PPI) network construction. In addition, 6 hub genes were extracted utilizing Cytoscape software for gene regulatory investigation, such as transcription factors (TFs)-gene network and miRNAs-gene network generation and immune landscape assessment. Finally, receiver operating characteristic (ROC) curves and candidate drugs prediction for SLE and DLBCL were performed based on the hub genes. Taken together, our study explored the molecular mechanisms by which EBV infection contribute to the susceptibility of DLBCL in SLE patients for the first time and identified potential biomarkers and therapeutic targets for patients with SLE and DLBCL.

## Results

### Identification of DEGs among EBV infection, SLE and DLBCL

**Figure 1:**
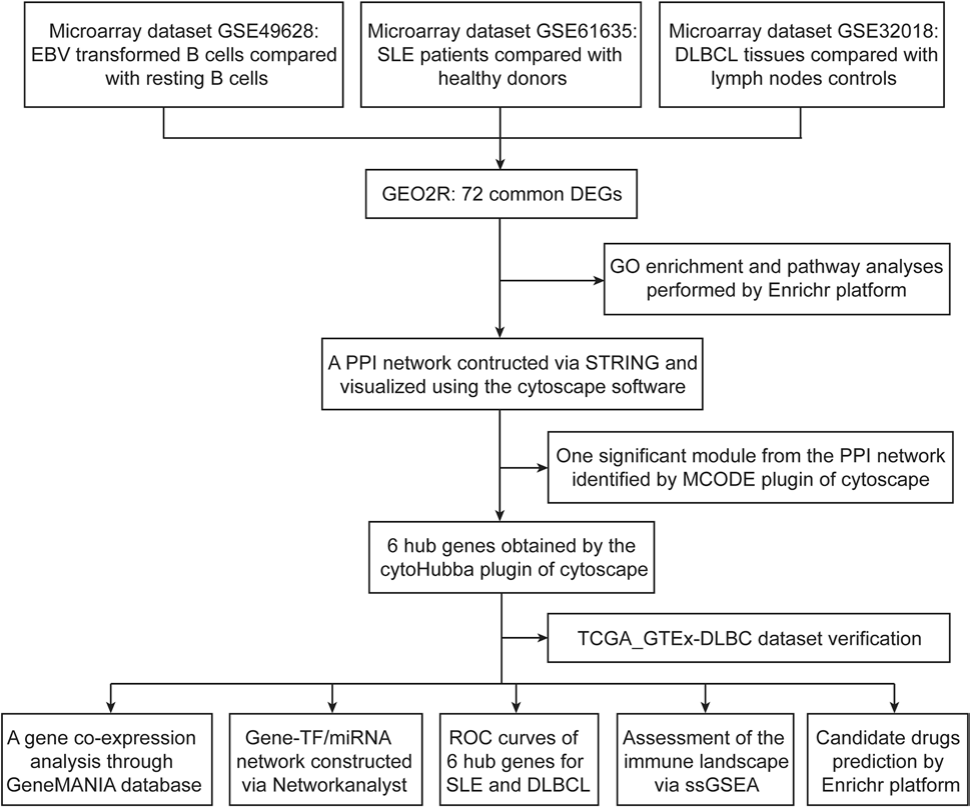
Flowchart for this investigation.

**Figure 2:**
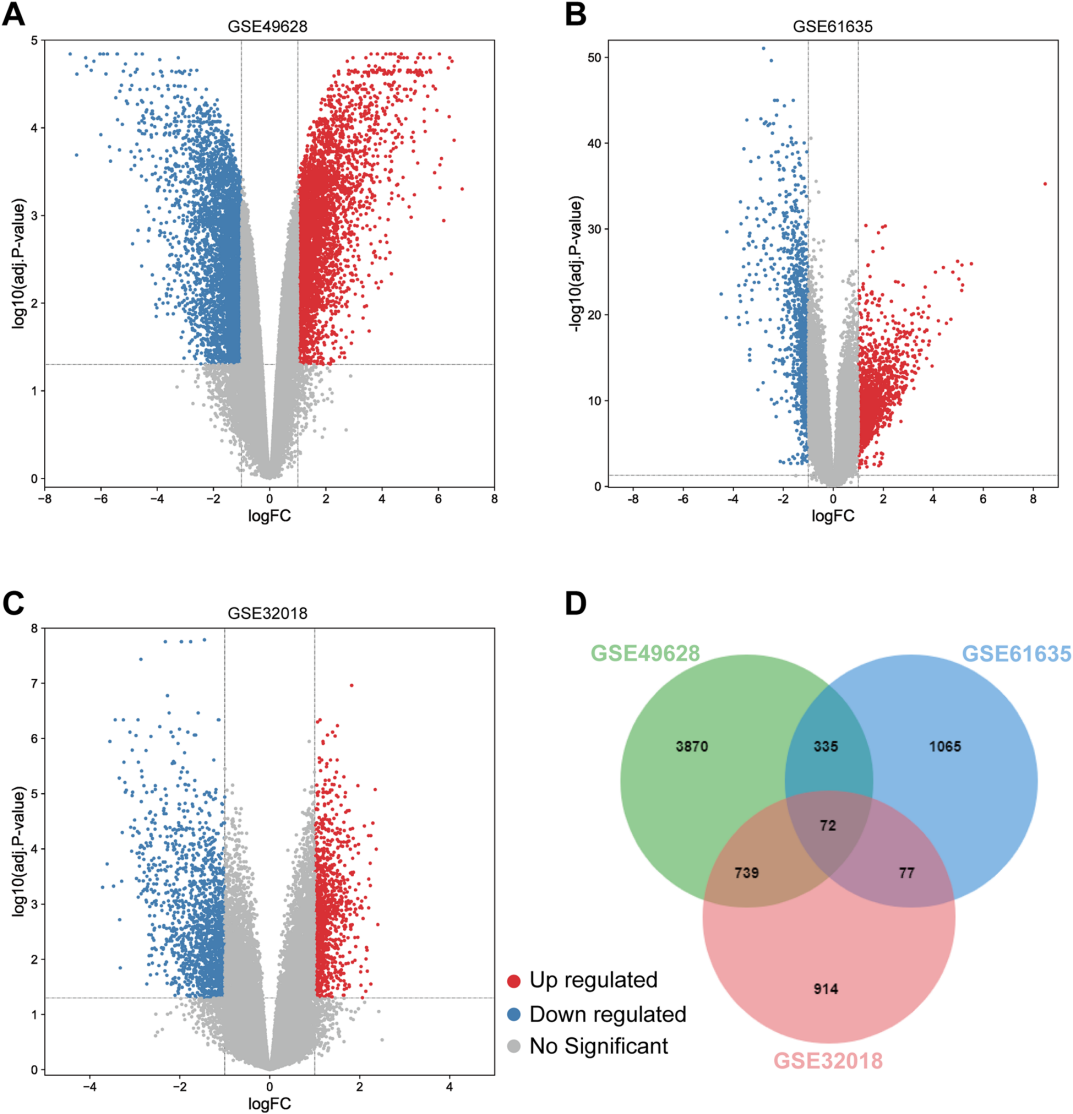
Volcano diagrams and Venn diagram. The volcano maps of GSE49628 (**A**), GSE61635 (**B**), and GSE32018 (**C**). Up-regulated genes are shown in red, while down-regulated genes are shown in blue. (**D**) There were 72 DEGs shared by these three datasets.

Figure 1 depicts the whole work flow of this project. Firstly, 5016 genes, comprising 2883 up-regulated and 2133 down-regulated genes from the GSE49628 dataset, were differentially expressed for EBV infection (Fig. 2A). A total of 1549 DEGs were discovered using the SLE dataset (GSE61635), of which 992 genes showed up-regulation and 557 genes showed down-regulation (Fig. 2B). We discovered 1802 DEGs for the DLBCL dataset (GSE32018), comprising 791 up-regulated genes and 1011 down-regulated genes (Fig. 2C). With the use of the cutoff criteria (*P*-value < 0.05 and |logFC|> 1), all significant DEGs were retrieved. The summarized information of these datasets was listed in Table 1. Then by taking the intersection of DEGs of EBV, SLE and DLBCL datasets, 72 common DEGs were identified and visualized by Venn diagrams (Fig. 2D).

**Table 1:** Summary of the datasets used in this investigation, together with their geo-features and quantitative metrics.

Disease name	GEO accession	GEO platform	Total DEGs count	Up regulated DEGs count	Down regulated DEGs count
EBV	GSE49628	GPL570	5016	2883	2133
SLE	GSE61635	GPL570	1549	992	557
DLBLC	GSE32018	GPL6480	1802	791	1011

### GO and pathway enrichment analysis

**Figure 3:**
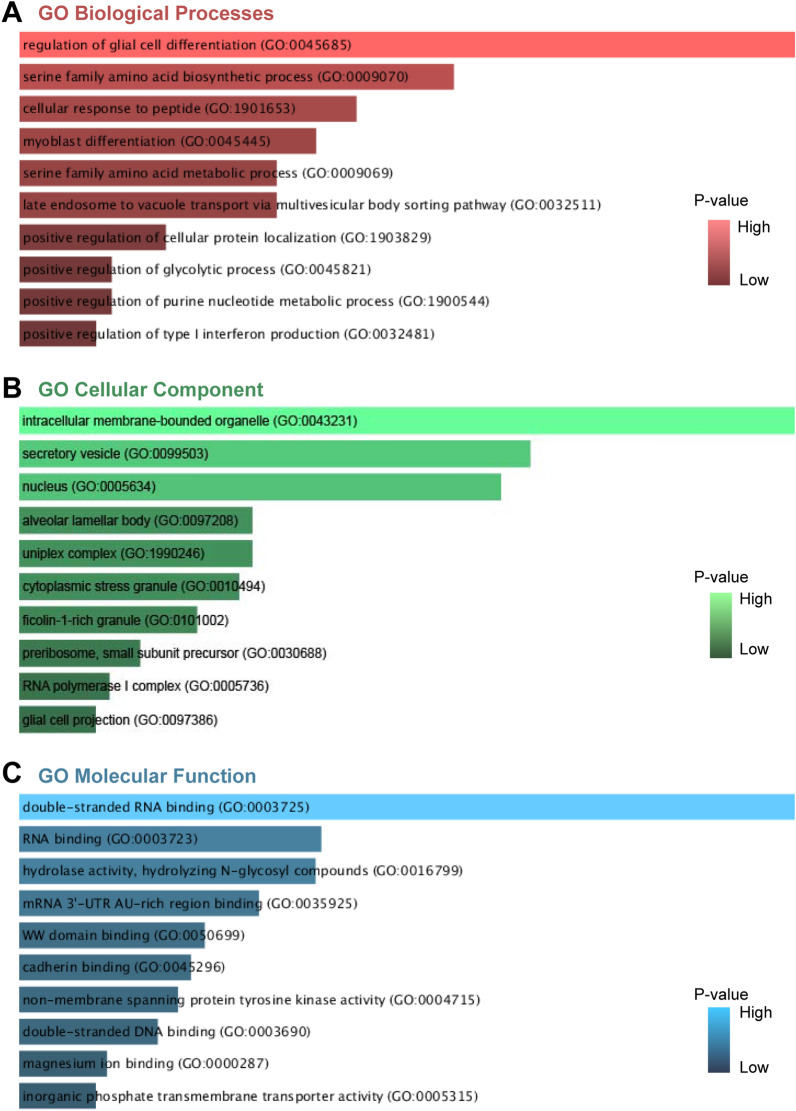
GO terms of common genes between EBV infection, SLE and DLBCL. (**A**) Biological Processes, (**B**) cellular component, (**C**) molecular function.

**Figure 4:**
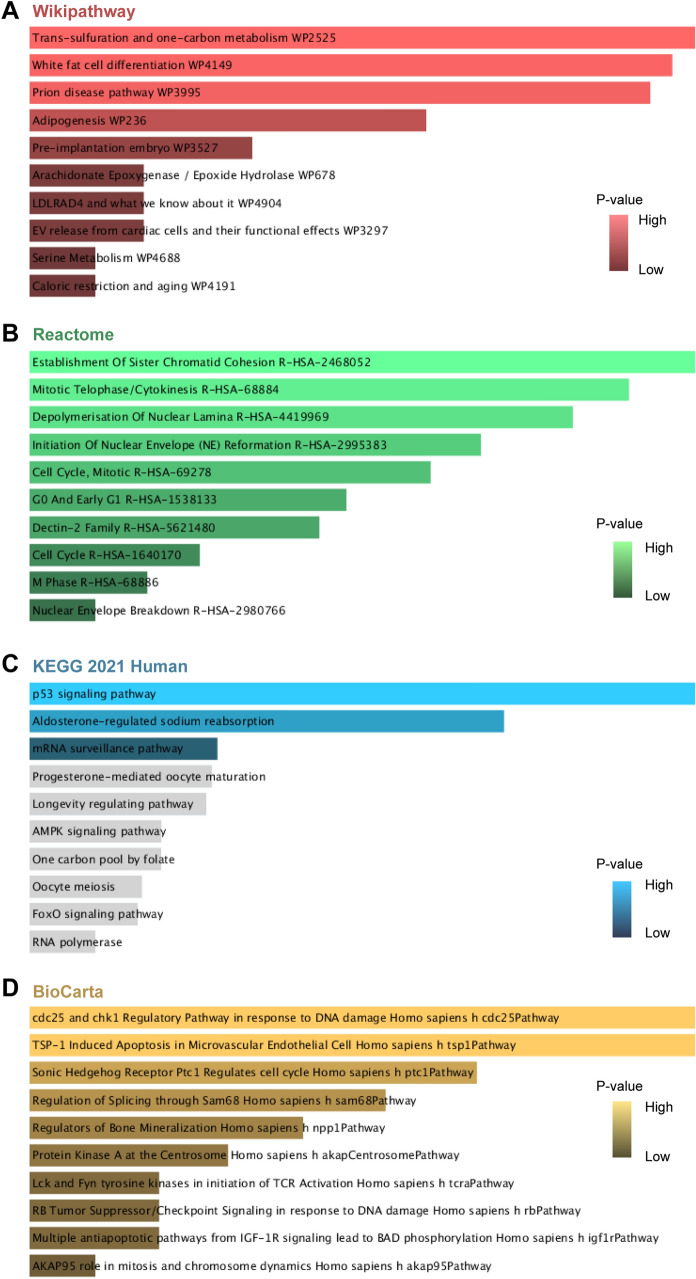
Pathway enrichment analysis of common genes between EBV infection, SLE and DLBCL. (**A**) Wikipathway, (**B**) Reactome Pathway, (**C**) KEGG Human Pathway, (**D**) BioCarta Pathway.

We used the Enrichr online tool to analyze GO and pathway enrichment of the common DEGs. The rank of significant terms was determined by the *P*-value. GO analysis consists of three categories, including biological process, cellular component and molecular function. The top 10 significant terms of different categories were summarized in Table 2 and listed as bar graphs in Fig. 3. The most impacted pathways of the common DEGs among EBV infection, SLE and DLBCL were identified from four databases (WikiPathways, Reactome, KEGG and BioCarta). The top 10 pathways gathered from the above datasets were listed in Table 3 and also shown precisely in bar graphs in Fig. 4.

**Table 2:** Ontological analysis of shared DEGs between EBV, SLE, and DLBCL.

Category	GO ID	Term	*P*-Values	Genes
GO Biological process	GO:0045685	Regulation of glial cell differentiation	2.65E-04	CDK1;TNFRSF21
	GO:0009070	Serine family amino acid biosynthetic process	8.24E-04	MTHFD1;PSPH
	GO:1901653	Cellular response to peptide	0.001138615	FYN;IGF1;CHMP5
	GO:0045445	Myoblast differentiation	0.001301854	MBNL1;IGF1
	GO:0009069	Serine family amino acid metabolic process	0.001484374	MTHFD1;PSPH
	GO:0032511	Late endosome to vacuole transport via multivesicular body sorting pathway	0.001484374	LEPROT;CHMP5
	GO:1903829	Positive regulation of cellular protein localization	0.002145527	CDK1;FYN;BICD1
	GO:0045821	Positive regulation of glycolytic process	0.002567618	ZBTB20;IGF1
	GO:1900544	Positive regulation of purine nucleotide metabolic process	0.002567618	ZBTB20;IGF1
	GO:0032481	Positive regulation of type I interferon production	0.002704008	ZBP1;RIOK3;LRRFIP1
GO Cellular Component	GO:0043231	Intracellular membrane-bounded organelle	0.001192748	TOP2A;NEK2;TMPO;KIF23;NEIL3DEPDC1;CDK1
	GO:0099503	Secretory vesicle	0.004872687	IGF1;BICD1
	GO:0005634	Nucleus	0.005698888	TOP2A;NEK2;TMPO;KIF23;NEIL3;DEPDC1;CDK1
	GO:0097208	Alveolar lamellar body	0.021409026	LAMP3
	GO:1990246	Uniplex complex	0.021409026	MICU3
	GO:0010494	Cytoplasmic stress granule	0.022972237	MBNL1;ELAVL1
	GO:0101002	Ficolin-1-rich granule	0.028757131	CLEC4C;CAND1;TNFAIP6
	GO:0030688	Preribosome, small subunit precursor	0.038904184	RIOK3
	GO:0005736	RNA polymerase I complex	0.045815566	POLR1E
	GO:0097386	Glial cell projection	0.049252856	FYN
GO Molecular function	GO:0003725	Double-stranded RNA binding	6.52064E-06	ZBP1;MBNL1;STRBP;LRRFIP1;ELAVL1
	GO:0003723	RNA binding	0.001391275	TOP2A;ZBP1;MBNL1;STRBP;MRPS23;MSI2;ELAVL1
	GO:0016799	Hydrolase activity, hydrolyzing *N*-glycosyl compounds	0.001484374	NEIL3;MACROD2
	GO:0035925	mRNA 3'-UTR AU-rich region binding	0.002817823	ELAVL1;RBMS3
	GO:0050699	WW domain binding	0.005208639	PRRG4;PMEPA1
	GO:0045296	Cadherin binding	0.006086267	PPFIBP1;LRRFIP1;RPL15;TMPO;CHMP5
	GO:0004715	Non-membrane spanning protein tyrosine kinase activity	0.007042367	FYN;FRK
	GO:0003690	Double-stranded DNA binding	0.008866374	ZBP1;KLF12;NEIL3;ZBTB20;BACH2;KLF2;NR3C2
	GO:0000287	Magnesium ion binding	0.015749168	TOP2A;EPHX2;PSPH
	GO:0005315	Inorganic phosphate transmembrane transporter activity	0.017872498	ANKH

**Table 3:** Pathway enrichment analysis of shared DEGs between EBV, SLE, and DLBCL.

Category	Pathways	*P*-Values	Genes
WikiPathways	Trans-sulfuration and one-carbon metabolism WP2525	0.005554958	MTHFD1;PSPH
	White fat cell differentiation WP4149	0.005911567	EBF1;KLF2
	Prion disease pathway WP3995	0.006278388	EBF1;FYN
	Adipogenesis WP236	0.011549909	MBNL1;EBF1;IGF1
	Pre-implantation embryo WP3527	0.018552642	ELAVL1;NR3C2
	Arachidonate Epoxygenase / Epoxide Hydrolase WP678	0.024932997	EPHX2
	LDLRAD4 and what we know about it WP4904	0.024932997	PMEPA1
	EV release from cardiac cells and their functional effects WP3297	0.024932997	KLF2
	Serine Metabolism WP4688	0.028444455	PSPH
	Caloric restriction and aging WP4191	0.028444455	IGF1
Reactome	Establishment Of Sister Chromatid Cohesion R-HSA-2468052	6.88E-04	PMEPA1;ESCO2
	Mitotic Telophase/Cytokinesis R-HSA-68884	9.72E-04	PMEPA1;KIF23
	Depolymerisation Of Nuclear Lamina R-HSA-4419969	0.001301854	CDK1;TMPO
	Initiation Of Nuclear Envelope (NE) Reformation R-HSA-2995383	0.002100521	CDK1;TMPO
	Cell Cycle, Mitotic R-HSA-69278	0.002729699	TOP2A;CDK1;PMEPA1;NEK2;KIF23;ESCO2
	G0 And Early G1 R-HSA-1538133	0.004232191	TOP2A;CDK1
	Dectin-2 Family R-HSA-5621480	0.004872687	CLEC4C;FYN
	Cell Cycle R-HSA-1640170	0.009080403	TOP2A;CDK1;PMEPA1;NEK2;KIF23;ESCO2
	M Phase R-HSA-68886	0.011934571	CDK1;PMEPA1;NEK2;KIF23;TMPO
	Nuclear Envelope Breakdown R-HSA-2980766	0.015645345	CDK1;TMPO
KEGG 2021 Human	p53 signaling pathway	0.002322805	SESN3;CDK1;IGF1
	Aldosterone-regulated sodium reabsorption	0.007846291	IGF1;NR3C2
	mRNA surveillance pathway	0.04867657	MSI2;GSPT1
	Progesterone-mediated oocyte maturation	0.050463783	CDK1;IGF1
	Longevity regulating pathway	0.052274479	SESN3;IGF1
	AMPK signaling pathway	0.069559815	IGF1;ELAVL1
	One carbon pool by folate	0.069621662	MTHFD1
	Oocyte meiosis	0.078812459	CDK1;IGF1
	FoxO signaling pathway	0.080918832	IGF1;KLF2
	RNA polymerase	0.105852996	POLR1E
BioCarta	cdc25 and chk1 Regulatory Pathway in response to DNA damage Homo sapiens h cdc25Pathway	0.024932997	CDK1
	TSP-1 Induced Apoptosis in Microvascular Endothelial Cell Homo sapiens h tsp1Pathway	0.024932997	FYN
	Sonic Hedgehog Receptor Ptc1 Regulates cell cycle Homo sapiens h ptc1Pathway	0.031943443	CDK1
	Regulation of Splicing through Sam68 Homo sapiens h sam68Pathway	0.035430005	CDK1
	Regulators of Bone Mineralization Homo sapiens h npp1Pathway	0.038904184	ANKH
	Protein Kinase A at the Centrosome Homo sapiens h akapCentrosomePathway	0.042366023	CDK1
	Lck and Fyn tyrosine kinases in initiation of TCR Activation Homo sapiens h tcraPathway	0.045815566	FYN
	RB Tumor Suppressor/Checkpoint Signaling in response to DNA damage Homo sapiens h rbPathway	0.045815566	CDK1
	Multiple antiapoptotic pathways from IGF-1R signaling lead to BAD phosphorylation Homo sapiens h igf1rPathway	0.045815566	IGF1
	AKAP95 role in mitosis and chromosome dynamics Homo sapiens h akap95Pathway	0.049252856	CDK1

### PPI network and submodule analysis

**Figure 5:**
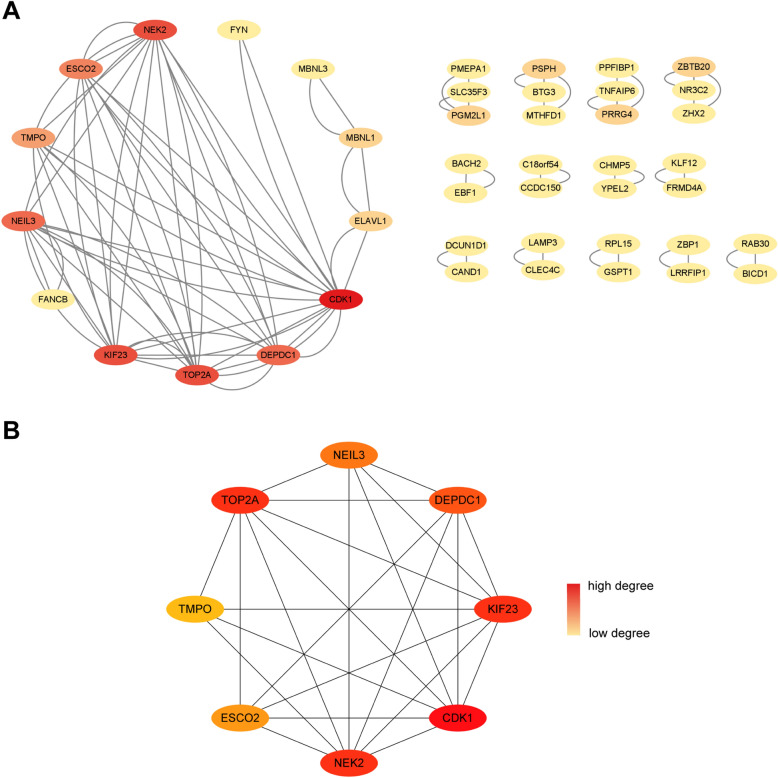
PPI network and gene module analysis. (**A**) PPI network diagram. (**B**) Significant gene module. The redder the color of the gene in the network, the higher the connectivity of the gene with other genes.

The PPI network of 72 common DEGs was analyzed by STRING platform, and the result was further visualized with Cytoscape software. The 42 nodes and 91 edges of the PPI network were shown in Fig. 5A. The stronger the connection of the gene with other genes, the redder the node was in the PPI network. Besides, we applied the MCODE plug-in of Cytoscape to construct a key gene module, which contained 8 common DEGs (Fig. 5B).

### Hub gene identification and functional analysis

**Figure 6:**
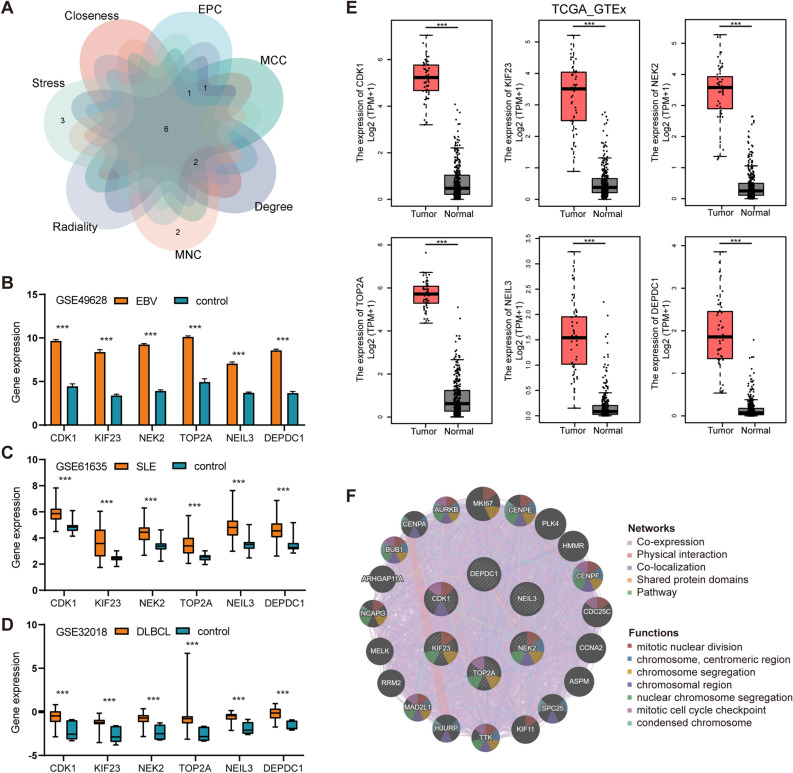
Venn diagram and hub gene co-expression network. (**A**) The Venn diagram displayed six hub genes that were filtered by seven algorithms. (**B**) Differential expression of hub genes in EBV-transformed B cells and resting B cells in the GSE49628 dataset. (**C**) Differential expression of hub genes in SLE patients and healthy donors in the GSE61635 dataset. (**D**) Differential expression of hub genes between DLBCL samples and healthy controls in the GSE32018 dataset. (**E**) Differential expression of hub genes between DLBCL samples and healthy controls in the TCGA_GTEx-DLBC dataset. (**F**) GeneMANIA was used to assess hub genes and the co-expressed genes. *P < 0.05, **P < 0.01, and ***P < 0.001.

Using 7 algorithms of cytoHubba plug-in of Cytoscape, the top 10 hub genes were screened. By applying the intersection of Venn diagrams, we finally identified 6 common hub genes, including CDK1, KIF23, NEK2, TOP2A, NEIL3 and DEPDC1 (Fig. 6A). The expression of the six hub genes were further verified in the EBV infection dataset (GSE49628), SLE dataset (GSE61635) and DLBCL datasets (GSE32018 and TCGA_GTEx-DLBC) (Fig. 6B–E). All of the six genes expressions were higher in EBV infection, SLE and DLBCL group compared to the control group. Through the GeneMANIA database, a complex gene interaction network was constructed for understanding the biological roles of the common hub genes, with the co-expression of 92.3%, co-localization of 3.98%, physical interactions of 3.52%, pathway of 0.12% and shared protein domains of 0.08% (Fig. 6F). Based on the 6 hub genes, 20 related genes were identified, showing that they were mainly associated with mitotic nuclear division, chromosome segregation and cell cycle checkpoint. Besides, GO and pathway enrichment of the hub genes was analyzed, showing the similar results from DEGs (Supplementary Figs. 2 and 3).

### Determination of regulatory signatures

**Figure 7:**
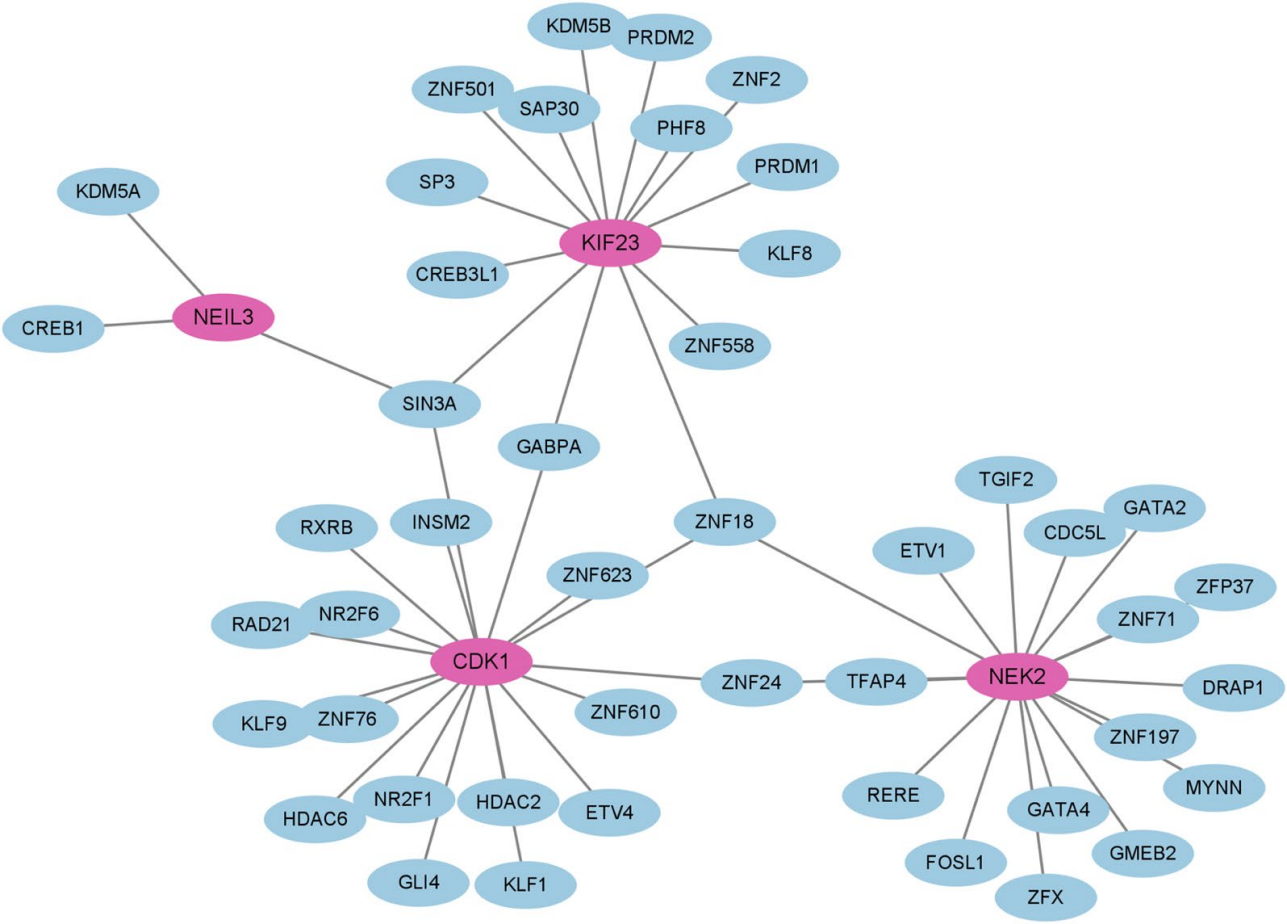
The TFs-gene regulatory network. Hub genes are represented by the pink nodes, and TF genes are represented by the other blue nodes.

**Figure 8:**
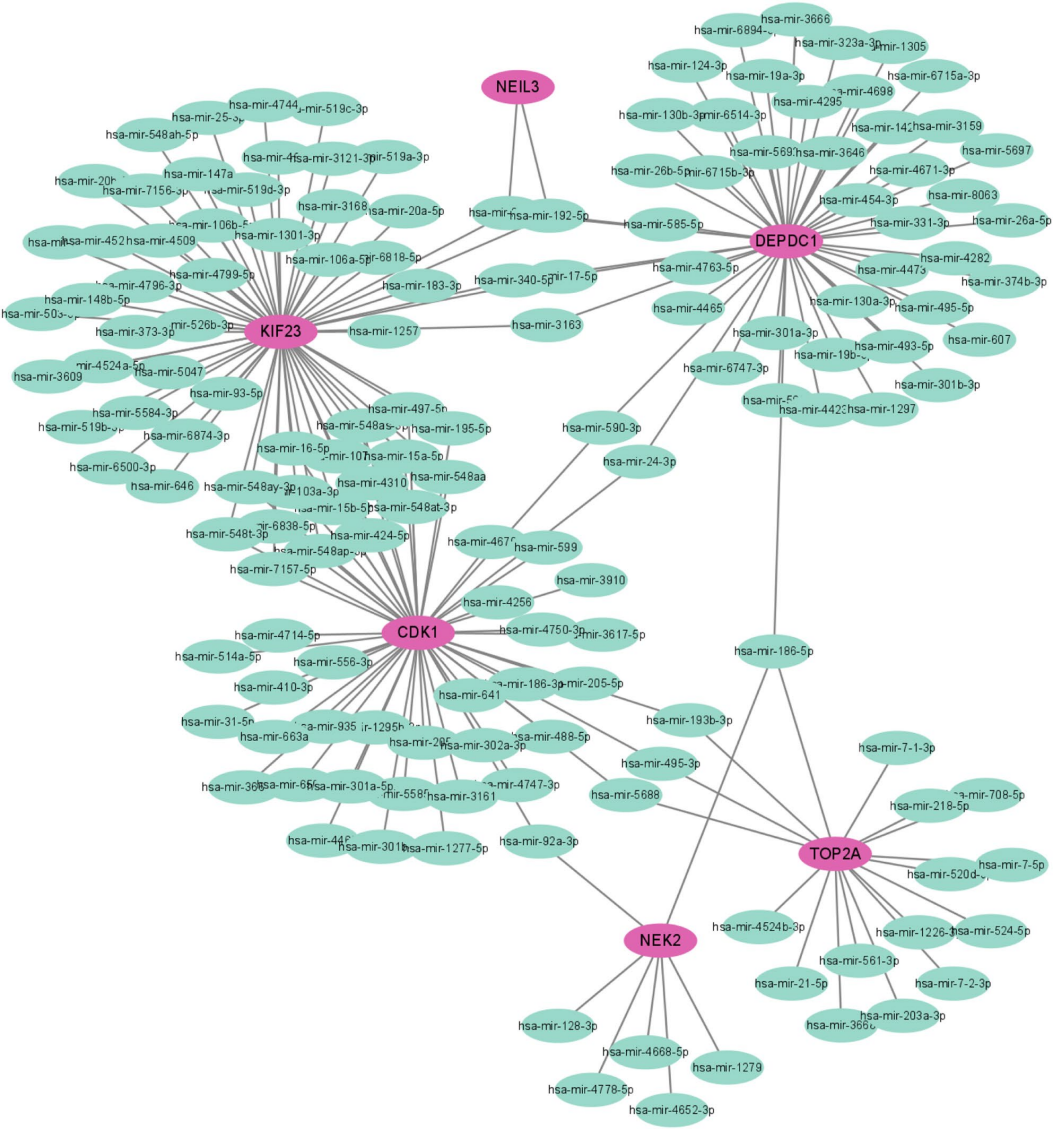
The miRNAs-gene regulatory network. Hub genes are represented by the pink nodes, and miRNAs are represented by the other green nodes.

To identify the regulatory molecules of hub genes at the transcriptional level, we constructed TFs-gene and miRNAs-gene networks using NetworkAnalyst platform, which were visualized by Cytoscape. As shown in Fig. 7, the TFs-gene interaction network contains 50 nodes and 52 edges. CDK1 was modulated by 18 TF genes, and NEK2 was modulated by 17 TF genes. The TFs such as SIN3A, GABPA, ZNF18 and ZNF24 regulated many hub genes in the network. The miRNA-gene regulatory network was also predicted by Networkanalyst and created by Cytoscape, including159 nodes and 185 edges (Fig. 8). It has been ascertained that 30 miRNAs regulated with more than one hub gene, which demonstrated the high interaction between them.

### ROC curves of hub genes

**Figure 9:**
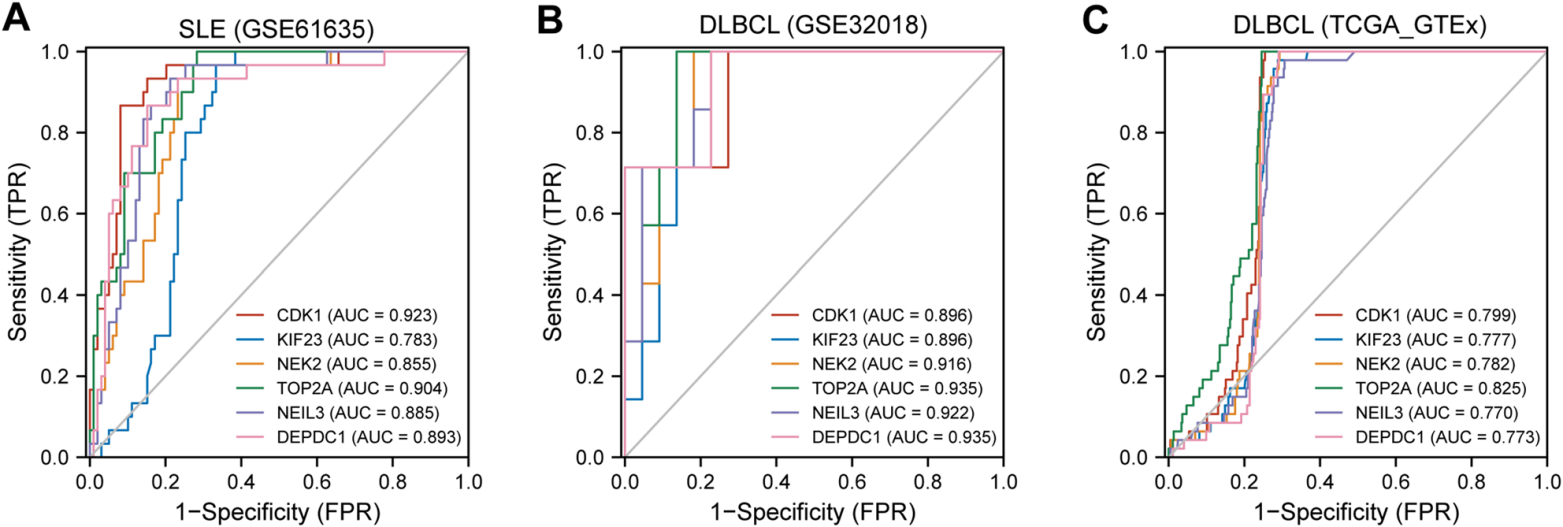
Verification of common diagnostic biomarkers. (**A**) The ROC curve used to verify the diagnostic efficacy in GSE61635. (**B**) The ROC curve used to verify the diagnostic efficacy in GSE32018. (**C**) The ROC curve used to verify the diagnostic efficacy in TCGA_GTEx dataset.

The diagnostic effectiveness of the 6 hub genes was evaluated using ROC curves. To identify SLE patients from healthy controls in the SLE dataset (GSE61635), CDK1 (AUC: 0.923) and TOP2A (AUC: 0.904) showed preferable diagnostic effectiveness (Fig. 9A). For separating DLBCL patients from healthy controls in the DLBCL dataset (GSE32018), TOP2A (AUC: 0.935), DEPDC1 (AUC: 0.935), NEIL3 (AUC: 0.922), and NEK2 (AUC: 0.916) showed good diagnostic performance (Fig. 9B). To distinguish DLBCL patients from healthy controls in the TCGA_GETx dataset (Fig. 9C), TOP2A (AUC: 0.825), CDK1 (AUC: 0.799) and NEK2 (AUC: 0.782) ranked the top 3 genes.

### Immune infiltration assessment

**Figure 10:**
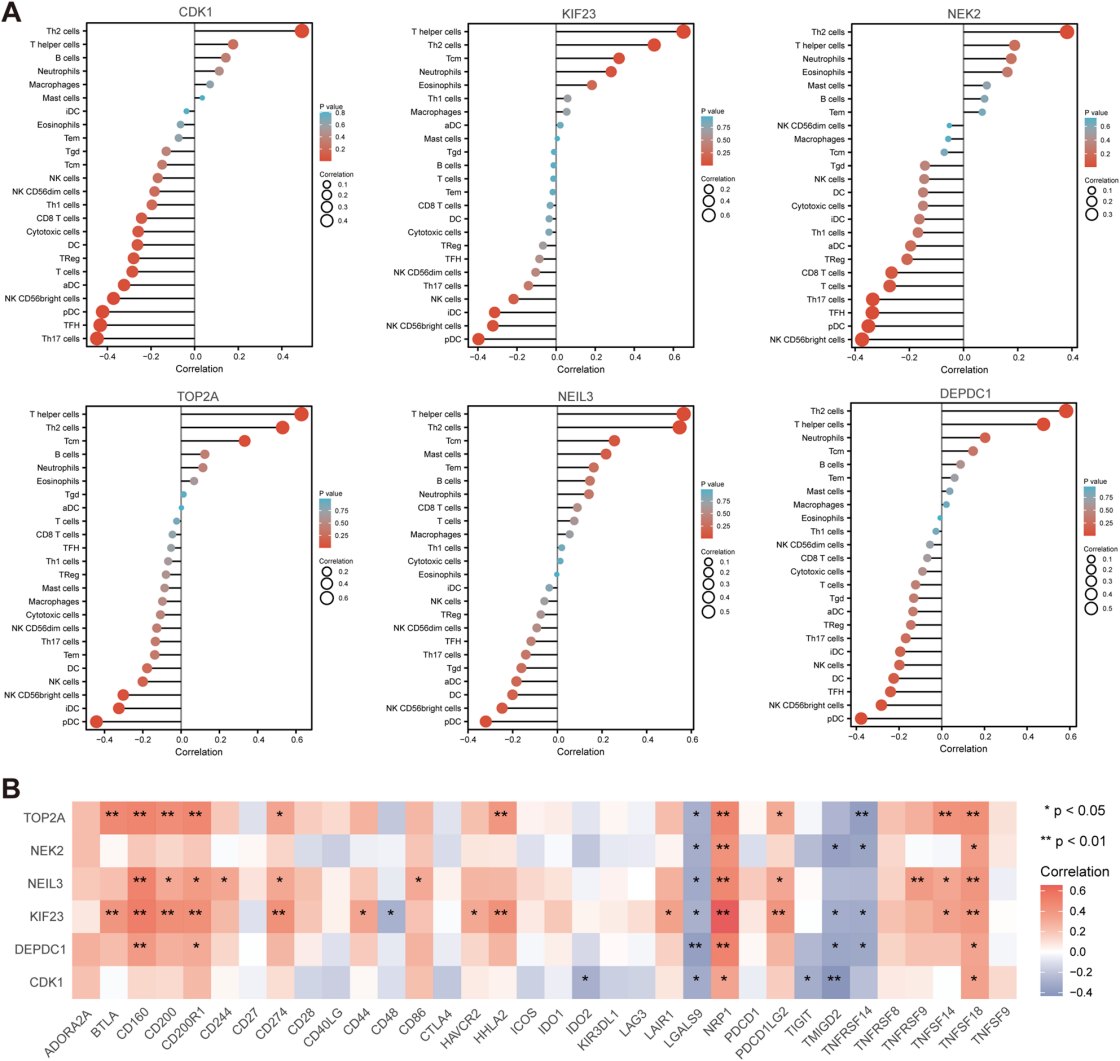
Immune landscape assessment. (**A**) Relationship of hub genes expression and immune cell subtypes in DLBCL patients. (**B**) Heatmap of the correlation between hub genes expression and immune checkpoint. *P < 0.05, **P < 0.01, and ***P < 0.001.

We investigated the relationship between the expression of hub genes and the infiltration of immune cells in DLBCL based on the TIMER database (Fig. 10A). The results showed that the expressions of CDK1, KIF23, NEK2, TOP2A, NEIL3 and DEPDC1 were all positively associated with T helper 2 (Th2) cells and T helper cells. On the contrary, the expressions of these six hub genes were negatively linked with plasmacytoid dendritic cells (pDCs) and NK CD56bright cells. In addition, immune checkpoint correlation analysis showed that hub genes were closely associated with various immune checkpoint factors, such as NRP1, TNFSF18 and LGALS9 (Fig. 10B).

### Prediction of candidate drugs

Using the Enrichr platform, which is based on the DSigDB database, the top 10 candidate therapeutic compounds were extracted and sorted by their *P*-value in the areas of hub genes as prospective pharmacological targets for EBV infection, SLE, and DLBCL (Table 4). It was discovered that the three pharmacological molecules that interacted with the majority of genes were testosterone (CTD 00006844), resveratrol (CTD 00002483), and calcitriol (CTD 00005558).

**Table 4:** Prediction of top 10 candidate drugs for EBV, SLE, and DLBCL.

Name of drugs	*P*-Values	Genes
LUCANTHONE CTD 00006227	7.96E−10	TOP2A;CDK1;DEPDC1;KIF23;NEK2
testosterone CTD 00006844	5.84E−08	TOP2A;NEIL3;CDK1;DEPDC1;KIF23;NEK2
trifluridine MCF7 DOWN	8.16E−08	TOP2A;DEPDC1;KIF23
monobenzone PC3 DOWN	1.55E−07	TOP2A;DEPDC1;KIF23;NEK2
0173570–0000 PC3 DOWN	1.84E−07	DEPDC1;KIF23;NEK2
troglitazone CTD 00002415	2.12E−07	TOP2A;NEIL3;CDK1;KIF23;NEK2
resveratrol CTD 00002483	2.62E−07	TOP2A;NEIL3;CDK1;DEPDC1;KIF23;NEK2
methotrexate MCF7 DOWN	3.30E−07	TOP2A;DEPDC1;KIF23
ciclopirox MCF7 DOWN	7.13E−07	TOP2A;DEPDC1;KIF23
calcitriol CTD 00005558	8.77E−07	TOP2A;NEIL3;CDK1;DEPDC1;KIF23;NEK2

## Discussion

Several studies have indicated that EBV infections could trigger the occurrence of SLE and DLBCL. Additionally, SLE patients are more likely to develop DLBCL, suggesting that EBV infection may be the link between SLE and DLBCL. However, the underlying mechanism is still unclear. In this study, we performed a series of bioinformatics analysis and attempted to reveal the possible molecular machanisms by which EBV infection contribute to the pathogenesis of DLBCL in SLE patients for the first time and discovered potential biomarkers and therapeutic targets for SLE and DLBCL.

GO analysis was performed based on the 72 common DEGs identified by the three datasets of EBV infection, SLE and DLBCL. In terms of biological process terms, regulation of glial cell differentiation, serine family amino acid biosynthetic process, cellular response to peptide, myoblast differentiation and serine family amino acid metabolic process were the most significant. Serine is a significant supply of one-carbon units, which are necessary building blocks for the production of nucleotides. EBV upregulates import and synthesis of serine through its encoded protein EBNA2, which is essential for EBV-driven B cell survival and proliferation^22^. Moreover, reducing the amount of extracellular serine available or preventing the production of serine from glycolytic intermediates have also been proposed as innovative therapeutic approaches for the treatment of autoimmune diseases^23^ and B-cell lymphomas^24^. Regarding the molecular function, double-stranded RNA binding, hydrolase activity, cadherin binding were the top terms. EBER-1 and EBER-2, two short RNAs that EBV encodes, are highly expressed in latently infected cells. EBERs can bind to the double-stranded RNA-activated protein kinase (PKR) and prevent it from being phosphorylated, leading to the resistance of IFN-alpha-induced apoptosis in EBV-positive lymphoma^25^. As for cellular components, intracellular membrane-bounded organelle, secretory vesicle and nucleus ranked the top 3. Secretory vesicles released from EBV-infected cells are capable of promoting inflammation and immune dysfunction, which may contribute to autoimmune diseases and numerous malignancies^26^.

The KEGG pathway enrichment analysis showed that p53 signaling pathway, aldosterone-regulated sodium reabsorption and mRNA surveillance pathway are the top 3 significant pathways. Mutations that inactivate p53 promote genomic instability and are hallmark of cancer^27^. EBV-encoded oncoprotein latent membrane protein 1 (LMP1) is reported to promote the growth of lymphoma cells by the degradation of p53^28^. Interestingly, mutations in p53 have been shown to increase autoimmune susceptibility in multiple strains of mice^29,30^. The autoantibodies against p53 have been detected in the serums of patients with SLE, which could functionally block p53 activation and affect apoptosis^31^. Results from WikiPathways indicated that trans-sulfuration and one-carbon metabolism, white fat cell differentiation and prion disease pathway are the most significant. The trans-sulfuration route is a biochemical process that connects methionine metabolism to the production of cellular redox-controlling molecules, which contributes to atherosclerosis and tumor development^32^. Reactome analysis showed that the most interacted gene pathways are establishment of sister chromatid cohesion, mitotic telophase/cytokinesis and depolymerisat of nuclear lamina. According to recent research, abnormal sister chromatid cohesion leads to chromosomal instability, which in turn promotes the growth of cancer^33^. As for BioCarta, the top pathways are as follows: cdc25 and chk1 regulatory pathway in response to DNA damage, TSP-1 induced apoptosis in microvascular endothelial cell and sonic hedgehog receptor Ptc1 regulates cell cycle.

Based on the PPI network, CDK1, KIF23, NEK2, TOP2A, NEIL3 and DEPDC1 were selected as hub genes. CDK1 is able to regulate cell cycle progression and transcription^34^. CDK1 expression was up-regulated in EBV-positive DLBCL and nasal natural killer/T-cell lymphoma (NNKTL). Treatment with CDK1 inhibitors causes the death of EBV-transformed cells^35–37^. In addition, type I interferon (IFN) signaling is also thought to be a major pathogenic route in SLE. The excessively increased type I IFN signaling in SLE may be caused by overexpression of CDK1. KIF23, a kinesin 6 family member that is found at the interzone of mitotic spindles, is essential for cytokinesis^38^. KIF23 expression is increased in DLBCL and is a risk factor for this disease^39^. NEK2, a member of NIMA-related kinase family that regulates cell cycle, is up-regulated in a variety of malignancies, including DLBCL^40,41^. TOP2A is a multifunctional nuclear enzyme required during DNA replication, transcription and DNA damage repair^42^. It was identified that TOP2A-nucleolin interaction is essential for regulating Top2A targeting agent induced DLBCL cell death^43^. TOP2A is also proposed as potential biomarker for SLE diagnosis by comparative analysis^44^. For the remaining two hub genes, NEIL3 and DEPDC1, there are no publications describing their role in EBV, SLE or DLBCL, which highlights its significance for future research.

We also performed the TFs-gene and miRNAs-gene connection to discover the transcriptional and post-transcriptional regulators of the hub genes. The TFs such as SIN3A and ZNF18 regulated most hub genes in the network. SIN3A is hypothesized to control gene expression by acting as histone deacetylases, which is linked to tumor progression^45^. EBV nuclear antigen 3C is reported to recruit SIN3A to repress CDKN2A, which is important for immortal human B-lymphoblastoid cell line proliferation^46^. Furthermore, three miRNAs, including hsa-miRNA-186, hsa-miRNA-192 and hsa-miRNA-215 were identified to regulate most hub genes. These miRNAs play critical role in several types of cancers, such as breast cancer, gastric cancer and colorectal cancer^47–49^. However, their function in SLE or DLBCL has not been reported.

With the ROC curves, we found that six hub genes exhibit good diagnostic performance in SLE and DLBCL. Furthermore, we used a cohort of DLBCL patients from TCGA dataset to investigate the association between the expression levels of the six hub genes and overall survival. By ultilizing GEPIA2^50^ tool, Kaplan–Meier survival analysis and Cox proportional hazards regression to assess the association between gene expression and patient survival were conducted (Supplementary Fig. 1). However, we observed that the expression levels of these hub genes were not associated with overall survival in DLBCL patients. These findings suggest that while these hub genes may be useful for diagnostic purposes, they may not be reliable prognostic markers for DLBCL.

With assessment of immune infiltration, we observed that hub genes expressions are positively correlated with Th2 cell infiltration and negatively linked with pDC in DLBCL. Th2 cells have been primarily associated with promoting tumor growth and suppressing anti-tumor immunity^51^, while pDCs can contribute to anti-tumor immunity by promoting the activation and expansion of effector immune cells, and by producing type I interferons^52^. In addition, we found that these 6 hub genes had the highest positive correlation with NRP1 expression. NRP1 can promote tumor angiogenesis, tumor cell migration and invasion, and the infiltration of immunosuppressive cells into the tumor microenvironment^53,54^. This finding suggested that hub genes may play a role in remodeling immune landscape of tumor microenvironment (TME) to promote DLBCL.

Six hub genes were applied to the DSigDB database for the prediction of potential medicines. The top 3 chemical molecules were listed as follow: lucanthone, testosterone and trifluridine. Recently, it was discovered that the anti-schistosomal drug lucanthone, which may pass the blood–brain barrier, inhibit autophagy and suppress the growth of breast cancer and glioblastoma^55,56^. In peripheral blood mononuclear cells from SLE patients, testosterone, one of the male hormones, is able to reduce the production of anti-DNA antibodies by inhibiting B cell hyperactivity, supporting the therapeutic effects for SLE^57,58^. Trifluridine, a thymidine-based nucleoside analog, is a novel oral cytotoxic chemotherapy licensed for the treatment of metastatic colorectal cancer refractory to standard therapies^59^. It is also an antiviral agent for topical use in the eye, which could effectively inhibit the replication of herpes simplex virus type 1^60^.

Certainly, our study has some limitations. On the one hand, there was a lack of information on patients with DLBCL secondary to SLE with EBV infection to further verify our finding.. On the other hand, our findings were obtained by pure bioinformatics analysis, so the function of hub genes and prospective medicines is required to be further confirmed by scientific investigation in *vitro* and in vivo.

## Conclusion

To the best of our knowledge, our study is the first to reveal shared DEGs, GO and pathway enrichment and PPI network for EBV infection, SLE and DLBCL using bioinformatic analysis to explore the potential molecular mechanisms underlying the contribution of EBV infection to the development of DLBCL in SLE patients. Our research also identified immune-related biomarkers and future therapeutic targets for patients with SLE and DLBCL, which will help better manage SLE patients and provide early diagnosis and treatment for DLBCL.

## Methods

### Compilation of datasets

GEO (www.ncbi.nlm.nih.gov/geo) is a big database providing gene expression profiles for a variety of disorders. It is free of charge and publicly available^61^. GSE49628^62^ dataset contains resting and EBV transformed B cells from 3 donors and GSE61635 dataset consists of 79 SLE patients and 30 healthy donors, which were both sequenced using the Affymetrix Human Genome U133 Plus 2.0 Array platform. GSE32018^63^ is of 21 DLBCL samples and 7 healthy controls, which was sequenced by Agilent-014850 Whole Human Genome Microarray 4 × 44 K G4112F platform. Gene expression data and patient clinical information were downloaded from TCGA database (project ID: TCGA_GTEx-DLBC) (https://portal.gdc.cancer.gov/). In total, information for 41 DLBCL tissues and 447 adjacent normal tissues was obtained.

### Identification of shared DEGs between EBV infection, SLE and DLBCL

An online program called GEO2R (www.ncbi.nlm.nih.gov/geo/geo2r/) can be used in the comparison and analysis between the groups with different gene expression^64^. GEO2R was used to identify DEGs for GSE49628, GSE61635 and GSE32018. DEGs were defined as genes with |log_2_ fold change (log_2_FC)|> 1.0 and an adjusted *P*-value < 0.05. The volcano map of DEGs from each dataset and the Venn diagram of shared DEGs between these three datasets were both plotted by Bioinformatics (https://www.bioinformatics.com.cn).

### Investigation of GO and pathways enrichment

In order to find the functions of the shared DEGs or hub genes in EBV infection, SLE and DLBCL, we utilized Enrichr^65^, a useful gene set enrichment online platform (https://maayanlab.cloud/Enrichr/), to conduct a series of enrichment analyses. Biological process, cellular component, and molecular function are the three elements of GO^66^. The shared pathways among DEGs or hub genes were identified based on four databases, including WikiPathways^67^, Reactome^68^, Kyoto Encyclopedia of Genes and Genomes (KEGG)^69–72^, and BioCarta^73^.

### PPI network construction and module analysis

The assessment of PPI network is the cornerstone of cellular biology for understanding protein function and the mechanism of cellular machinery operations. Search Tool for the Retrieval of Interacting Genes (STRING, http://string-db.org) is a database for studying PPI network with physical and functional interactions^74^. We used STRING to create the PPI network of shared DEGs with an interaction score greater than 0.4 and displayed it by Cytoscape (Version 3.9.1)^75^. The core functional module was produced using a Cytoscape plug-in—Molecular Complex Detection (MCODE)^76^.

### Recognition of hub genes

The hub genes of this study were identified using CytoHubba^77^, a plug-in of Cytoscape. Subsequently, the final hub genes were confirmed by seven algorithms (MCC, MNC, EPC, Closeness, Degree, Radiality and Stress) and visualized by Venn diagram. Based on the final hub genes, co-expression networks were constructed by GeneMANIA (http://genemania.org), an online platform for gene interactions prediction^78^.

### Validation of the hub genes in EBV infection, SLE and DLBCL

To confirm hub shared genes in EBV infection, SLE and DLBCL, we conducted the differentially expressed gene analysis on validation datasets (GSE49628, GSE61635 and GSE32018). GEPIA2^50^ (http://gepia2.cancer-pku.cn/#index) is an analysis tool containing RNA sequence expression data of tumors and normal tissue samples. We used GEPIA2 to analyze TCGA and GTEx databases of gene expression profiles in DLBCL. Unpaired comparisons of hub gene expression between two groups were analyzed by Wilcoxon rank-sum test. We also performed prognostic analysis of hub genes in DLBCL with the “survival plots” module using a Kaplan–Meier curve with GEPIA2.

### Identification of TFs and miRNAs interactions with hub genes

Proteins called TFs control how quickly genes are transcribed by binding to certain DNA regions. TFs-gene networks were created by utilizing ENCODE database^79^ on NetworkAnalyst platform (https://www.networkanalyst.ca/)^80^. Furthermore, miRNAs are a group of short non-coding RNAs, which can impede translation or degrade the target mRNA. The network of miRNAs-gene was acquired from mirTarbase^81^ via NetworkAnalyst. Cytoscape was used to display TFs-gene and miRNAs-gene regulatory networks.

### Receiver operating characteristic curves of hub genes

Receiver operating characteristic (ROC) curves of the hub genes on SLE (GSE61635) and DLBCL (GSE32018 and TCGA_GTEx-DLBC) were both plotted by Bioinformatics platform. The diagnostic ability of each hub gene was assessed using the calculation of area under ROC curve (AUC).

### Assessment of the immune landscape

We used immune cell infiltration and gene expression data from the TIMER database^82^ to identify relationships between the expression of hub genes and immune cell abundance in DLBCL, and plotted bubble plots to show these results. Spearman’s correlation analysis was used to analyze the relationship between hub genes and a range of immune-related genes, such as immune checkpoint-associated genes and immune cell subpopulation-associated genes.

### Prediction of candidate drugs

It is important to evaluate protein-drug interactions in this research. On the basis of hub genes, drug molecules were obtained from Drug Signatures database (DsigDB)^83^ on Enrichr platform. *P*-value was used to rank the candidate medications from small to large.

### Copyright permission of KEGG

We have contacted Kanehisa Laboratories. We do not directly use these KEGG Pathway map “images” in the article, we need not obtain copyright permission of KEGG. However, they believe that we have written our article using their data, they kindly ask us to cite the following articles in it^69–72^.

## Supplementary Information

Supplementary Figures.

## Data Availability

The datasets used in this investigation are accessible through online repositories. The article contains information on the repository names and accession numbers.
