# Supplementary material for: Bioinformatics analysis of the pathogenic link between Epstein-Barr virus infection, systemic lupus erythematosus and diffuse large B cell lymphoma
Source: Sci Rep. 2023 Apr 18;13:6310. doi: 10.1038/s41598-023-33585-2 (PMC10113247; doi:10.1038/s41598-023-33585-2)
Supplement: Supplementary file 1 — Supplementary Figures. [file 41598_2023_33585_MOESM1_ESM.pdf]

Supplementary material

Bioinformatics analysis of the pathogenic link between Epstein-Barr virus infection, systemic lupus erythematosus and diffuse large B cell lymphoma

Qian-Ying Zhu[1*]

[1]Department of Laboratory Medicine, The Eighth Affiliated Hospital, Sun Yat-sen University, Shenzhen 518003, PR China.

[*]Correspondence: 452653694@qq.com

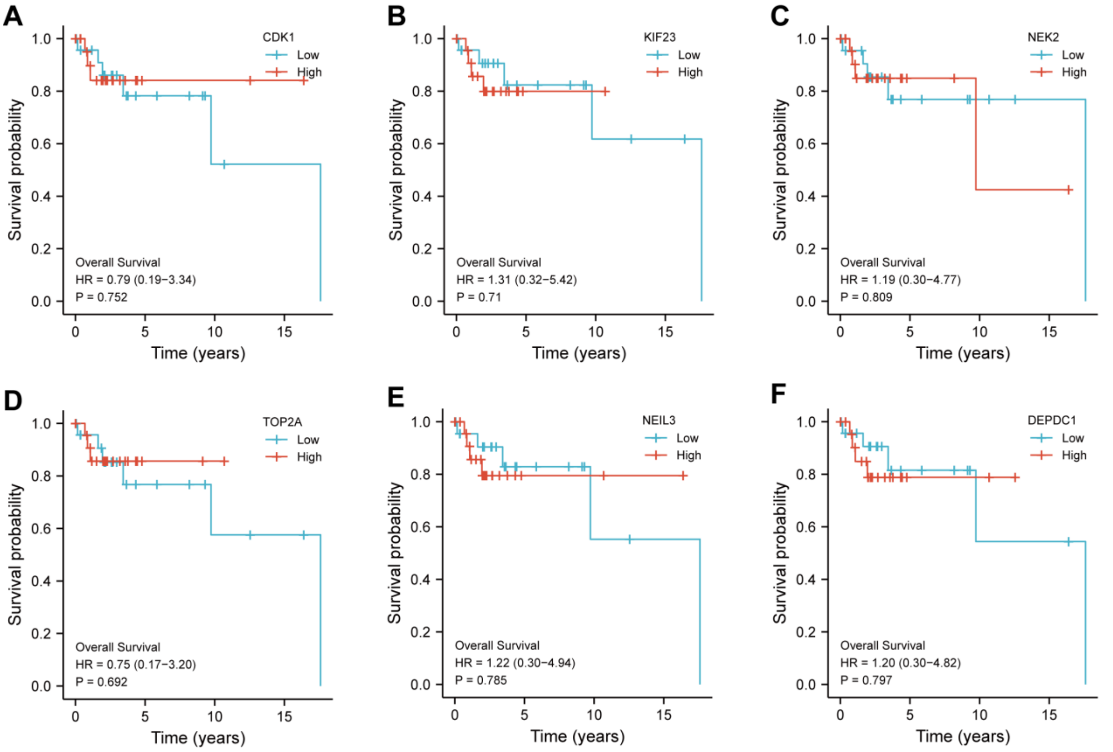

Supplementary figure 1. Kaplan–Meier curve of association of (A) CDK1, (B) KIF23, (C) NEK2, (D) TOP2A, (E) NEIL3, (F) DEPDC1 expression and DLBCL patients' overall survival in TCGA_GTEx-DLBC dataset.

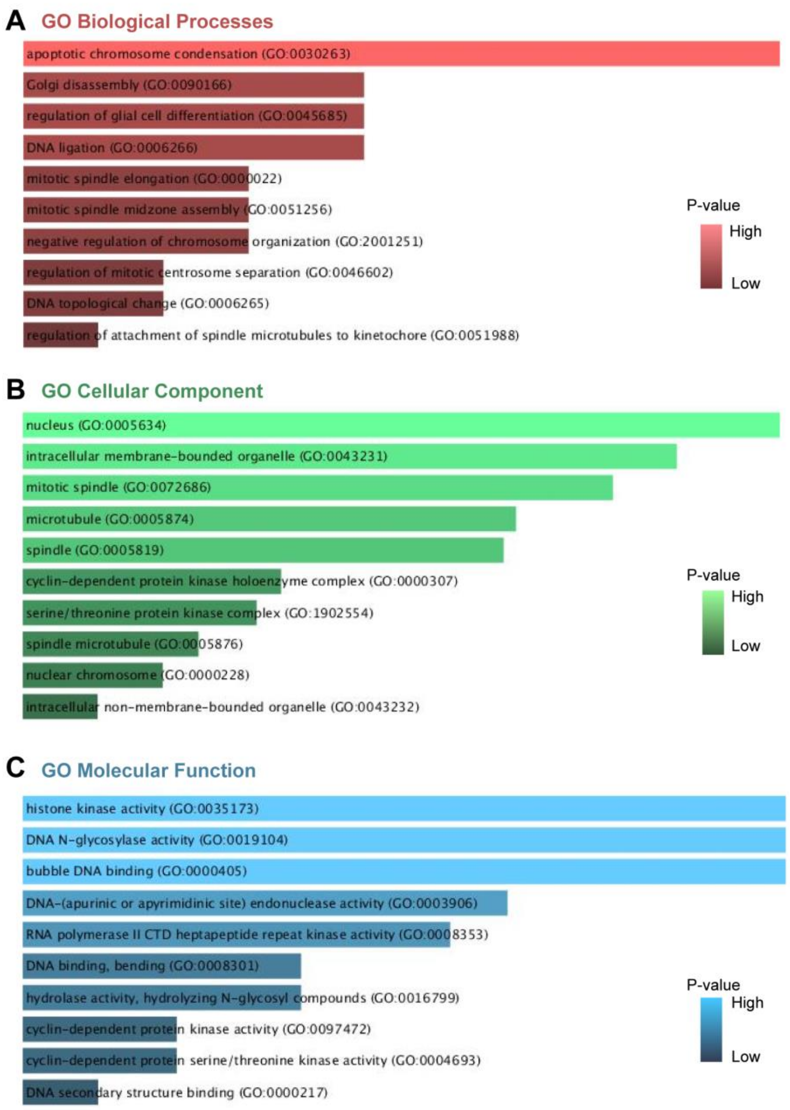

Supplementary figure 2. GO terms of hub genes between EBV infection, SLE and DLBCL. (A) Biological Processes, (B) Cellular Component, (C) Molecular Function.

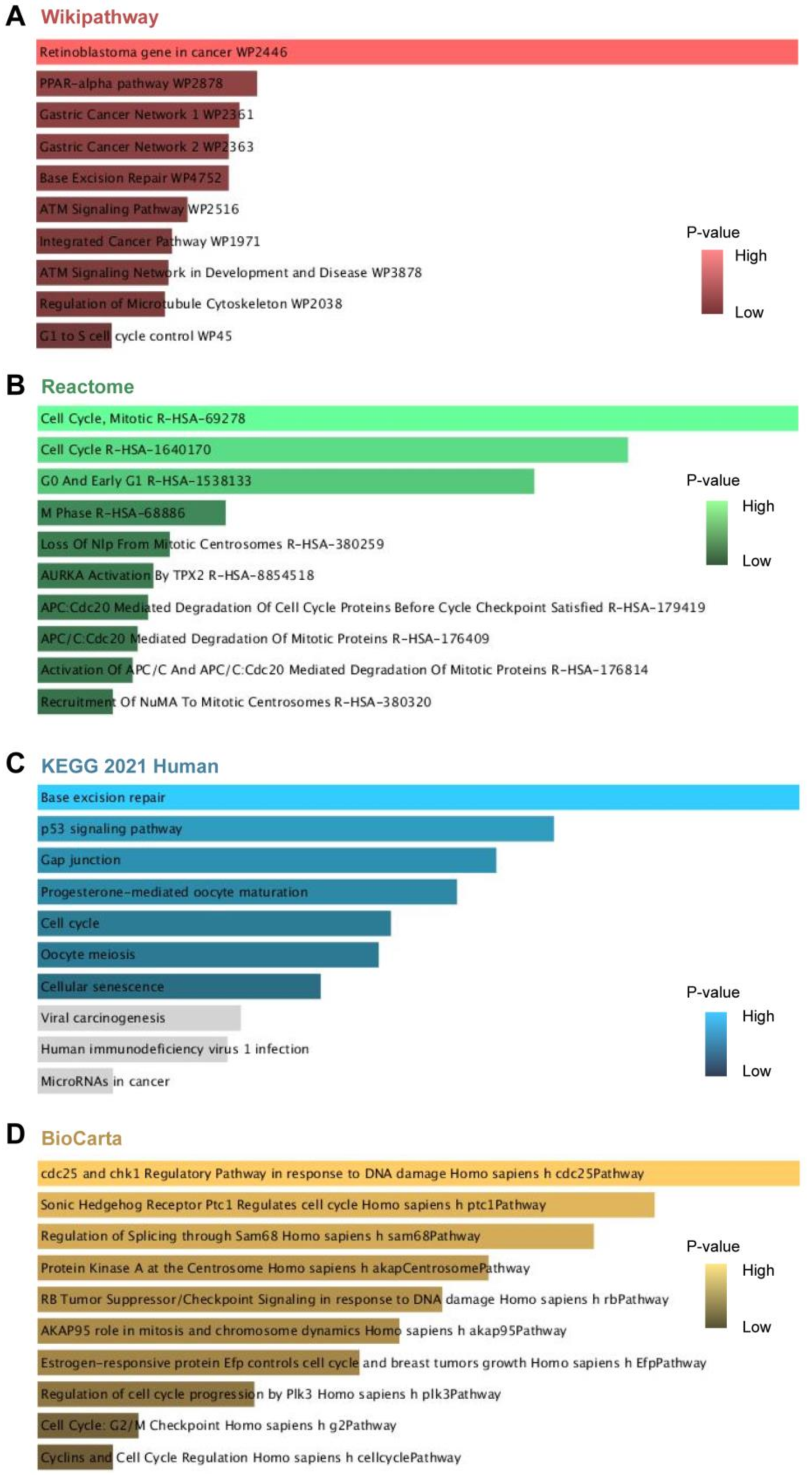

Supplementary figure 3. Pathway enrichment analysis of hub genes between EBV infection, SLE and DLBCL. (A) Wikipathway, (B) Reactome Pathway, (C) KEGG Human Pathway, (D) BioCarta Pathway.
